# Dental students’ perception of their educational environment in relation to their satisfaction with dentistry major: a cross-sectional study

**DOI:** 10.1186/s12909-023-04485-w

**Published:** 2023-07-17

**Authors:** Yoon Min Gil, Ji Soo Hong, Ju Ly Ban, Jae-Sung Kwon, Jae-Il Lee

**Affiliations:** 1 Department of Dentistry, School of Dentistry, Seoul National University, Seoul, South Korea; 2 Korean Institute of Dental Education and Evaluation, Seoul, South Korea; 3 CUNY Graduate Center, City University of New York, New York, USA; 4 Department and Research Institute of Dental Biomaterials and Bioengineering, BK21 FOUR Project, Yonsei University College of Dentistry, Seoul, South Korea; 5 Department of Oral Pathology, School of Dentistry and Dental Research Institute, Seoul National University, Seoul, South Korea

**Keywords:** Educational environment, Academic major satisfaction, Dundee ready education environment measure, Dental education

## Abstract

**Background:**

Students’ perception of their educational environment and satisfaction with their major can reveal the extent of their readiness to practice their profession after graduation. This study aimed to evaluate dental students’ perception of their educational environment and satisfaction with their major in dentistry, as well as the relationship between these two factors.

**Methods:**

An online survey was conducted in 2022 among first- to fourth-year students across 11 dental schools in Korea. The Dundee Ready Education Environment Measure (DREEM) and Academic Major Satisfaction Scale (AMSS) were used to measure the students’ perception of the educational environment and satisfaction with their major in dentistry, respectively.

**Results:**

A total of 539 students participated in the survey (response rate = 18.1%). The overall mean scores of the DREEM and AMSS were 125.03 (maximum score 200) and 22.01 (maximum score 30), respectively. Fourth-year students had the lowest scores in the overall DREEM, DREEM subscales (excluding students’ perceptions of atmosphere), and AMSS. The overall DREEM scores and all DREEM subscales showed statistically significant positive and moderate correlations with AMSS (*p* < 0.001).

**Conclusion:**

Using the DREEM, we identified areas that need improvement and the academic year (fourth year) that require proactive support. Considering the positive correlation between all DREEM subscales and the AMSS, measures to comprehensively improve the educational environment are needed to improve dental students’ satisfaction with their major.

## Background

In most countries, students are admitted to dental school through a highly competitive process. From the moment of admission, they learn numerous subjects and interact with peers, faculties, and patients in formal, informal, and hidden curriculums, ultimately becoming independent dentists upon graduation. These processes take place in an educational environment. Educational environment not only refers to the physical facilities and resources of an educational institution but also includes the psychosocial context that affects learning and teaching [1]. As the educational environment has a significant impact on students from admission to graduation, there is a sustained interest in evaluating and improving it in health professions education [1, 2].

Evaluating the educational environment offers several advantages [3]. Conducting evaluation during a specific time allows stakeholders to understand the state of the educational environment at the time and identify areas that need improvement. In the case of reforming the existing curriculum, the impact can be identified by comparing how the educational environment is perceived before and after the reform [4]. Evaluating the educational environment using the same tools with different groups of participants provides opportunities to compare results with other schools globally and reveal differences in perceptions between students and faculties [5]. Furthermore, many studies have been conducted to identify factors related to the educational environment [6] to help achieve desired goals through its improvement. For example, if the educational environment and student performance are found to be related, the educational institution can take steps to improve the environment to enhance student performance. The Dundee Ready Education Environment Measure (DREEM), which is a tool for evaluating the educational environment, has been widely used in various fields of health professions education such as medicine, dentistry, nursing, oriental medicine, and veterinary medicine [3, 7, 8]. In addition to evaluating the educational environment, many studies have used DREEM to identify factors related to the educational environment. As a result, it has been found that the educational environment affects academic achievement, quality of life, resilience, mindfulness, stress, and happiness [6, 9–11].

Academic majors in college or university such as dentistry, biology, or engineering can significantly impact students’ future career choices [12], and satisfaction is a key construct and evaluation index in the social sciences. Therefore, there is ongoing interest in and research on academic major satisfaction [13], which is a construct that determines whether college or university students are generally satisfied with their major [13]. In the context of dental education, this indicates dental students’ overall satisfaction with their major. Previous studies have revealed that academic major satisfaction is related to academic performance, career development, and life satisfaction [14–17]. Therefore, measuring students’ satisfaction with their majors can help understand their preparedness for future work. Moreover, as most students in health professions education work in the healthcare field after graduation, it is particularly important to ensure that they are satisfied with their majors.

Many studies have evaluated and compared the educational environment of dental schools in various countries [6, 18]; however, no study has evaluated the educational environments of Korean dental schools. Additionally, it has been difficult to find studies that investigate dental students’ satisfaction with their major in dentistry. Therefore, this study aimed to investigate Korean dental students’ perception of their educational environment and how this is related to their satisfaction with their major. This study will contribute to improving the educational environment and academic major satisfaction in dental schools.

## Methods

### Study design and settings

We conducted a cross-sectional study with students from 11 dental schools in Korea, consisting of 6 public and 5 private dental schools. While there may be slight variations in the curriculum among these dental schools, the first 2 years generally cover basic dental science, followed by 2 years of clinical dental education. An online survey was conducted among first to fourth-year dental students, excluding those in the pre-dental phase, to evaluate their perception of the educational environment and satisfaction with their dentistry major.

### Measures

#### Educational environment

The DREEM, which was developed to measure the basic medical educational environment, was used to identify participants’ perceptions of their educational environment [19]. The DREEM was used because it had been widely used in health professions education across many countries and had shown to be a reliable instrument [18]. The Korean version of the DREEM, which is used to measure the educational environment of Korean medical schools [20], was modified to fit the context of dental schools. One bilingual individual proficient in both Korean and English and two dental faculty members verified the modified version.

The DREEM consists of 50 items and 5 subscales. The five subscales are students’ perceptions of learning (SPL, 12 items), students’ perceptions of teachers (SPT, 11 items), students’ academic self-perceptions (SASP, 8 items), students’ perceptions of atmosphere (SPA, 12 items), and students’ social self-perceptions (SSSP, 7 items). Each item is rated on a 5-point Likert scale (0 = strongly disagree; 4 = strongly agree), and nine items are reverse-scored. Therefore, the maximum score for the overall DREEM is 200: 48 for SPL, 44 for SPT, 32 for SASP, 48 for SPA, and 28 for SSSP. Interpretation of the DREEM total score is as follows: 0–50 = very poor environment; 51–100 = plenty of problems; 101–150 = more positive than negative; 151–200 = excellent environment [21]. The score for each item can also be interpreted according to the following criteria: 3.5 or higher = educational aspects of excellence, 3.01–3.49 = positive educational aspects, 2.01–3.00 = improvable educational aspects, and 2.00 or less = problematic educational aspects. In this study, the internal reliability (Cronbach’s alpha) of the DREEM was 0.97, and the internal reliability of all DREEM subscales exceeded 0.70 (ranging from 0.74 to 0.90).

#### Academic major satisfaction

The Academic Major Satisfaction Scale (AMSS) developed by Nauta [13] and validated in Korean by Sovet et al. [22] was used to evaluate participants’ satisfaction with their major in dentistry. The AMSS consists of six items, each on a 5-point Likert scale (1 = strongly disagree; 5 = strongly agree), of which two are non-reverse-scored and four are reverse-scored. For example, the item “I often wish I hadn’t gotten into this major” is a reverse-scored item, and the item “Overall, I am happy with the major I’ve chosen” is a non-reverse-scored item. A higher total AMSS score indicates higher academic major satisfaction. In this study, the internal reliability of the AMSS was 0.81.

### Data collection

We surveyed 2,984 students (1,842 males and 1,142 females) comprising first- to fourth-year students across the 11 dental schools in Korea. The Korea Institute of Dental Education and Evaluation, which evaluates and accredits programs operated by Korean dental schools, obtained approval from each dental school to send an e-mail containing the Google survey link to their students. When the students accessed the link, they were provided with information about the purpose, meaning, anonymity, and voluntary nature of the study. The online survey was conducted from January to April 2022, and 539 dental students responded to the survey (response rate 18.1%).

### Sample size

Using G*Power version 3.1.9.7 [23], the required sample size was estimated. One-way analysis of variance (ANOVA) was selected for the statistical test. With a medium effect size of 0.25 [24], a significance level of 5%, a power of 95%, and a total of 4 groups, the estimated required sample size was determined to be 280 participants.

### Data analysis

Overall DREEM and DREEM subscales, as well as AMSS scores, were calculated by summing item scores for each test. Independent samples t-test and one-way ANOVA were used to compare each component score according to school type (public, private), gender (male, female), and academic year (first, second, third, fourth year). If there was a significant difference in the ANOVA analysis, Tukey’s honest significant difference test was performed for post hoc comparison. The *p-*value was considered statistically significant if it was less than 0.05.

The Pearson correlation coefficient was used to measure the correlation between the educational environment and academic major satisfaction. The following generally accepted correlation interpretation criteria were used: 0.00–0.01 is “negligible correlation,” 0.10–0.39 is “weak correlation,” 0.40–0.69 is “moderate correlation,” 0.70–0.89 is “strong correlation,” and 0.90–1.00 is “very strong correlation” [25]. IBM Statistical Package for the Social Sciences (IBM Corp., Armonk, NY, USA) was used for statistical analysis.

## Results

Of 2,984 dental students in Korea, 539 students comprising 306 males and 233 females responded to the questionnaire (response rate 18.1%). The demographic characteristics of the participants are shown in Table 1.

**Table 1:** Demographic characteristics of the participants

**Characteristic**	**n (%)**
School type	
Public	326 (60.5%)
Private	213 (39.5%)
Gender	
Male	306 (56.8%)
Female	233 (43.2%)
Academic year	
1st	167 (31.0%)
2nd	136 (25.2%)
3rd	95 (17.6%)
4th	141 (26.2%)

Table 2 shows the overall DREEM, DREEM subscales, and AMSS scores according to demographic characteristics. The mean score of the overall DREEM was 125.03. There was no significant difference in the overall DREEM, DREEM subscales, and AMSS scores between school types. In terms of gender, there was no significant difference between male and female students in the overall DREEM and DREEM subscales; however, female students had significantly higher AMSS scores (t = -2.245, *p* = 0.025). Regarding academic year, fourth-year students had the lowest scores in the overall DREEM, DREEM subscales excluding SPA, and AMSS.

**Table 2:** DREEM and AMSS scores by demographic characteristics

**Variables**		**SPL**	**SPT**	**SASP**	**SPA**	**SSSP**	**Overall DREEM**	**Overall AMSS**
All participants Mean (SD)		30.42 (8.01)	29.19 (6.78)	21.52 (5.01)	28.49 (8.42)	15.41 (4.02)	125.03 (29.53)	22.01 (4.36)
School Type Mean (SD)	Public	30.76 (8.11)	29.46 (6.89)	21.70 (5.09)	28.70 (8.46)	15.54 (4.07)	126.16 (29.75)	22.07 (4.61)
	Private	29.89 (7.83)	28.78 (6.60)	21.24 (4.89)	28.17 (8.38)	15.22 (3.95)	123.30 (29.18)	21.92 (3.95)
	t	1.239	1.132	1.024	0.714	0.923	1.099	0.425
	*p*-value	0.216	0.258	0.306	0.475	0.357	0.272	0.671
Gender Mean (SD)	Male	30.36 (8.42)	29.11 (6.96)	21.51 (5.47)	28.64 (9.12)	15.54 (4.35)	125.16 (31.69)	21.65 (4.55)
	Female	30.49 (7.45)	29.30 (6.56)	21.52 (4.35)	28.30 (7.42)	15.25 (3.56)	124.86 (26.50)	22.48 (4.06)
	t	-0.200	-0.308	-0.025	0.478	0.830	0.117	-2.245
	*p*-value	0.841	0.758	0.980	0.633	0.407	0.907	0.025
Academic Year Mean (SD)	1st	32.12 (8.23)	30.72 (7.08)	21.52 (5.62)	31.19 (8.35)	16.30 (4.14)	131.85 (31.03)	22.75 (4.56)
	2nd	31.38 (7.33)	29.65 (6.05)	22.06 (4.50)	28.58 (7.91)	15.77 (3.69)	127.44 (26.97)	22.58 (4.12)
	3rd	30.66 (7.77)	29.65 (6.58)	22.32 (4.85)	28.14 (8.26)	15.77 (4.05)	126.54 (27.95)	21.62 (4.63)
	4th	27.30 (7.72)	26.63 (6.59)	20.45 (4.69)	25.45 (8.11)	13.78 (3.73)	113.61 (28.10)	20.85 (3.90)
	F	10.908	10.411	3.497	12.671	11.717	10.959	6.119
	*p*-value	< .001	< .001	< .001	< .001	< .001	< .001	< .001
	Post-hoc	4 < 1,2,3	4 < 1,2,3	4 < 2,3	2,3,4 < 1 2 < 4	4 < 1,2,3	4 < 1,2,3	4 < 1,2

*DREEM* Dundee Ready Education Environment Measure, *AMSS* Academic Major Satisfaction Scale, *SPL* Students’ Perceptions of Learning; *SPT* Students’ Perceptions of Teachers, *SASP* Students’ Academic Self-Perceptions, *SPA* Students’ Perceptions of Atmosphere, *SSSP* Students’ Social Self-Perceptions

Table 3 presents the results of the analysis of individual items in the DREEM, separated by subscale. Seven items had an average score of 2.0 or less. Among them, three items were in SPA, and the four remaining items were in SPL, SPT, SASP, and SSSP, respectively.

**Table 3:** Problematic educational aspects

**Subscale**	**Item**	**Mean (SD)**
SPL	The teaching over-emphasizes factual learning	1.99 (1.08)
SPT	The teachers are authoritarian	1.99 (1.18)
SASP	I can memorize all I need	1.82 (1.05)
SPA	The atmosphere is relaxed during the clinical teaching	1.94 (1.06)
	I find the experience disappointing	1.81 (1.16)
	The enjoyment outweighs the stress of studying dentistry	1.99 (1.10)
SSSP	There is a good support system for students who get stressed	1.89 (1.20)

*SPL* Students’ Perceptions of Learning, *SPT* Students’ Perceptions of Teachers, *SASP* Students’ Academic Self-Perceptions, *SPA* Students’ Perceptions of Atmosphere, *SSSP* Students’ Social Self-Perceptions

As shown in Table 4, the correlation analysis revealed that educational environment and satisfaction with academic major had a positive and moderate correlation. The correlation coefficient between the overall DREEM and the AMSS was 0.525 (*p* < 0.001), indicating a moderate correlation between the two variables. Furthermore, significant positive correlations were found between all DREEM subscales and the AMSS. Specifically, the highest correlation was observed between SPT and AMSS (*r* = 0.557, *p* < 0.001), followed by SSSP (*r* = 0.524, *p* < 0.001), SPL (*r* = 0.459, *p* < 0.001), SPA (*r* = 0.446, *p* < 0.001), and SASP (*r* = 0.437, *p* < 0.001). Based on the generally accepted correlation interpretation criteria [25], these correlations can be considered moderate, suggesting a significant relationship between the variables.

**Table 4:** Correlations between DREEM and AMSS scores

	**SPL**	**SPT**	**SASP**	**SPA**	**SSSP**	**Overall DREEM**
AMSS	0.459	0.557	0.437	0.446	0.524	0.525
*p*-value	< 0.001	< 0.001	< 0.001	< 0.001	< 0.001	< 0.001

*DREEM* Dundee Ready Education Environment Measure, *AMSS* Academic Major Satisfaction Scale, *SPL* Students’ Perceptions of Learning, *SPT* Students’ Perceptions of Teachers, *SASP* Students’ Academic Self-Perceptions, *SPA* Students’ Perceptions of Atmosphere, *SSSP* Students’ Social Self-Perceptions

## Discussion

Using the DREEM and AMSS, Korean dental students’ perceptions of their educational environments and satisfaction with their major were measured. The overall mean score of the DREEM was 125.03, which is interpreted as “more positive than negative”; no significant difference was found based on gender (male, female) or school type (public, private). As the educational environments of most other dental schools around the world also fall under the “more positive than negative” score domain [26], this score for Korean dental schools was not comparatively lower. However, as problematic results were found regarding seven items of the DREEM, efforts to improve the educational environment are still needed. All five subscales of the DREEM (SPL, SPT, SASP, SPA, and SSSP) were found to be significantly correlated with the AMSS.

Among the seven items that received low mean scores and are considered problematic educational aspects, “The teachers are authoritarian” and “The teaching overemphasizes factual learning” are items with commonly low scores at dental schools in various countries [5, 27–30]. Faculties are experts whose authority is derived from their expertise and experience. In the master-apprentice relationship model, which has been a prominent feature of health professions education in the past, the hierarchical relationship between faculties and students is sometimes perceived as authoritarianism [31, 32]. While authority should be respected, the authoritarian relationship between school faculties and students needs to be ameliorated. Interestingly, some studies showed a difference in perception between faculties and students on the item “The teachers are authoritarian” [5]: compared to how students think of them, faculties tend to view themselves as less authoritarian. Faculties and students may have different perceptions of what an “authoritarian teacher” is because students tend to appreciate faculties who show passion and care more, while faculties tend to value expertise more highly [33]. The relationship between a faculty member and their student is not hierarchical and should go beyond simply transferring and receiving knowledge. A relationship of mutual respect and cooperation can help students develop a professional identity [34]; therefore, a culture where faculties accept students as partners and respect their opinions can yield a better educational environment [35].

Although traditional dental curricula emphasized memorizing considerable knowledge, current dental curricula have attempted to embrace a variety of educational methods to improve student engagement and motivation for learning [36]. However, this study’s results reveal that the traditional teacher-centered method, in which the faculty transmits considerable knowledge to the student, is still dominant. The explosive increase in biomedical knowledge and uncertainty of the current clinical situation has further exposed the limitations of the rote learning method [37]. Students in health professions education are expected to be independent experts with problem-solving and critical thinking competencies. Therefore, students should engage in self-directed learning, while schools should employ various educational methods based on contemporary educational theories [38]. For example, following the adult learning theory, which says that adult learners are more motivated when they study topics relevant to their future work [39], the curriculum should continue to pursue integrated learning with clinical relevance and experience. Reinforcing early clinical exposure is also a highly useful method for connecting theory and practice [40].

Fourth-year students had lowest scores in the overall DREEM and all DREEM subscales except SPA. To place this in the context of the Korean dental education system, students begin treating real patients in their third year and take the National Dental Licensing Examination in their fourth year. This academic burden could be one of the factors that lowers fourth-year students’ perception of the educational environment. In previous studies where students have a lower perception of the educational environment in the clinical phase compared to the non-clinical phase, the cause was attributed to burdens associated with patient care and examination [26, 27, 30, 41]. Dental students experience more workload stress, psychological distress, and burnout as they advance in their studies [42–44]. However, as the low score for the “There is a good support system for students who get stressed” item indicates, the support system for students is still lacking, which is a common issue in many dental schools [5, 28–30]. As students who experience academic or mental problems tend to rely on their peers and refrain from seeking help from professional advisors [45], a systematic and proactive support system should be established. Students’ happiness and quality of life are also educational goals to be pursued, which can be fostered with a good educational environment [11].

Although several studies have been conducted on job satisfaction among dentists [46], it was difficult to find studies on students’ satisfaction with a major in dentistry. This study evaluated dental students’ satisfaction with their major in dentistry using the AMSS, which resulted in an overall mean score of 22.01 (maximum score 30). Compared to other recent studies, the results of this study are similar to that of Sovet et al. [22] (mean score 21.75), but the scores obtained in this study are lower than those of Milsom and Coughlin [17] (mean score 26.29) and Schenkenfelder et al. [47] (mean score 24.24). More importantly, it is worth paying attention to the result indicating that fourth year students, who will soon become dentists, have the lowest academic major satisfaction. This, together with their low perception of the educational environment, reflects a particular difficulty in the fourth year. As academic major satisfaction is a factor related to academic persistence [48], a robust support system for fourth-year students is needed so that they can become full-fledged dentists with minimum resistance. According to Nauta [13], academic major satisfaction is a potential predictor of future job and life satisfaction. Therefore, if dental students are satisfied with their dentistry major in a good educational environment, they are more likely to provide good dental care to the public as dentists in the future.

A positive correlation between the educational environment and academic major satisfaction was also found. Consistent with previous studies in which the overall DREEM and all DREEM subscales were related to resilience [49] and quality of life [3], the scores of the overall DREEM and all DREEM subscales in this study showed a significant positive correlation with academic major satisfaction. These results suggest that academic major satisfaction is not guaranteed by improving only a single subscale of the DREEM, instead, it is necessary to provide a comprehensively good educational environment that will enhance dental students’ satisfaction with their major and their successful career in dentistry. The “problematic educational aspects” found in this study require further research and action. Furthermore, since a temporary evaluation alone cannot improve the overall educational environment, a continuous quality improvement system should be implemented, such as creating a culture that promotes participation in education and collaboration among stakeholders [50].

Despite the strengths of this cross-sectional study, there are still several limitations. First, although the number of recruited participants exceeded the minimum sample size requirement, the response rate was relatively low. While there is no scientifically established minimum response rate in surveys [51], the risk of bias due to nonresponse still exists at low response rates. Therefore, this should be taken into consideration when interpreting the results of this study. Second, it is difficult to identify changes in dental students' perceptions over time. Longitudinal studies with more participants are needed. Third, although this study identified areas of the educational environment that needed improvement, there might still be problems that were not identified in the survey. Health conditions, disabilities, interpersonal relationships, and gender issues are sensitive topics for students to discuss with others; therefore, considerate and in-depth qualitative research is required to supplement the findings of this study.

## Conclusions

This study identified educational environment areas in need of improvement and a particular academic year (fourth year) that requires proactive support. Moreover, we identified a positive correlation between educational environment and academic major satisfaction. Considering these findings, we can conclude that measures to comprehensively improve the educational environment are needed to improve dental students’ satisfaction with their major.

## Data Availability

The datasets used and/or analysed during the current study are available from the corresponding author on reasonable request.

## Abbreviations

DREEM: Dundee Ready Education Environment Measure
AMSS: Academic Major Satisfaction Scale
SPL: Students’ Perceptions of Learning
SPT: Students’ Perceptions of Teachers
SASP: Students’ Academic Self-Perceptions
SPA: Students’ Perceptions of Atmosphere
SSSP: Students’ Social Self-Perceptions
ANOVA: Analysis of variance
